# Delineating nonmotor symptoms in early Parkinson's disease and first‐degree relatives

**DOI:** 10.1002/mds.26281

**Published:** 2015-07-14

**Authors:** Fahd Baig, Michael Lawton, Michal Rolinski, Claudio Ruffmann, Kannan Nithi, Samuel G. Evetts, Hugo R. Fernandes, Yoav Ben‐Shlomo, Michele T.M Hu

**Affiliations:** ^1^ Oxford Parkinson's Disease CentreUniversity of OxfordOxfordUnited Kingdom; ^2^ Nuffield Department of Clinical NeurosciencesUniversity of OxfordOxfordUnited Kingdom; ^3^ School of Social and Community Medicine, University of BristolBristolUnited Kingdom; ^4^ Department of NeurologyNorthampton General Hospital NHS TrustNorthamptonUnited Kingdom

**Keywords:** Parkinson's disease, first‐degree relatives, nonmotor symptoms, quality of life, treatment

## Abstract

Nonmotor symptoms (NMS) are an important prodromal feature of Parkinson's disease (PD). However, their frequency, treatment rates, and impact on health‐related quality of life (HRQoL) in the early motor phase is unclear. Rates of NMS in enriched at‐risk populations, such as first‐degree PD relatives, have not been delineated. We assessed NMS in an early cohort of PD, first‐degree PD relatives and control subjects to address these questions. In total, 769 population‐ascertained PD subjects within 3.5 years of diagnosis, 98 first‐degree PD relatives, and 287 control subjects were assessed at baseline across the following NMS domains: (1) neuropsychiatric; (2) gastrointestinal; (3) sleep; (4) sensory; (5) autonomic; and (6) sexual. NMS were much more common in PD, compared to control subjects. More than half of the PD cases had hyposmia, pain, fatigue, sleep disturbance, or urinary dysfunction. NMS were more frequent in those with the postural instability gait difficulty phenotype, compared to the tremor dominant (mean total number of NMS 7.8 vs. 6.2; *P* < 0.001). PD cases had worse HRQoL scores than controls (odds ratio: 4.1; *P* < 0.001), with depression, anxiety, and pain being stronger drivers than motor scores. NMS were rarely treated in routine clinical practice. First‐degree PD relatives did not significantly differ in NMS, compared to controls, in this baseline study. NMS are common in early PD and more common in those with postural instability gait difficulty phenotype or on treatment. Despite their major impact on quality of life, NMS are usually under‐recognized and untreated. © 2015 The Authors. *Movement* Disorders published by Wiley Periodicals, Inc. on behalf of International Parkinson and Movement Disorder Society.

Parkinson's disease (PD) is a progressive multisystem disorder of the nervous system. It is characterized by its cardinal motor features and is known to cause a broad range of nonmotor symptoms (NMS). Careful characterization of these features should improve clinical care, monitoring of disease progression, and improve our understanding of disease evolution from the premotor to early motor phases.

Throughout the disease course, there is mounting evidence of the importance of NMS in health‐related quality of life (HRQoL).^1^, ^2^ NMS even seem to correlate with worsening HRQoL more than motor symptoms.^3^ Crucially, despite these symptoms being manageable and often treatable, they are frequently unrecognized and undertreated in the clinical setting.^4^ Changes in severity of NMS are reported with the use of medication^5^, ^6^ and specific motor subtypes, with important implications for treatment and underlying pathophysiology.^7^, ^8^

Multiple studies have shown that NMS are common in established PD, but less is known about the early and premotor phases. A significant proportion of patients with pathologically proven PD will initially present to health care services with NMS. These individuals are more likely to experience a delay in diagnosis and higher rates of misdiagnosis.^9^ To study this prodromal phase, we have recruited first‐degree relatives of PD subjects, unselected on the basis of genotype (e.g., presence of leucine‐rich repeat kinase 2 [LRRK2] or glucocerebrosidase [GBA] mutations), given that this group have an increased risk of developing PD compared to those without affected relatives.^10^

This study assesses the frequency and impact of NMS in a large, population‐ascertained cohort of early PD subjects, an at‐risk population of first‐degree relatives, and unaffected controls. It provides a hitherto unparalleled opportunity to compare the breadth of NMS across one of largest cohorts of well‐characterized individuals of its type worldwide.

## Patients and Methods

### Participants

Full details of the protocol have been described previously.^11^, ^12^ In brief, PD patients diagnosed within 3.5 years were recruited between September 2010 and September 2014. Cases were eligible for inclusion if they met the UK PD Brain Bank criteria for diagnosis.^13^ The control population were recruited from spouses and friends of patients taking part in the study, as well as the general public. The at‐risk group was comprised of participants with a first‐degree relative carrying a diagnosis of PD. Data from the baseline study visit, and not longitudinal assessment, were included for analysis. Additional information on recruitment and exclusion criteria is included in Supporting Table 1.

**Table 1:** Basic demographics of all included participants stratified by subject group

Basic Demographics	PD (n = 769)	At‐Risk (PD Relatives) (n = 98)	Controls (n = 287)
Age, mean, range (SD)	67.7, 32‐89 (9.5)	59.8, 35‐86 (10.7)	65.3, 28‐88 (10.0)
Gender, female n (%)	261 (33.9)	57 (58.2)	150 (52.3)
Ethnicity, nonwhite n (%)	11 (1.4)	2 (2.1)	6 (2.1)
Age of motoric symptom onset, mean, range (SD)	64.8, 28‐87 (9.7)	n/a	n/a
Disease duration from symptom onset in years, mean, range (SD)	2.9, 0.2‐13.9 (1.9)	n/a	n/a
Disease duration from diagnosis in years, mean, (SD)	1.3 (1.0)	n/a	n/a
MDS‐UPDRS III, mean (SD)	26.4 (11.0)	2.4 (3.4)	1.7 (2.7)
H & Y stage, n (%)			
0	0		
1	178 (23.2)		
2	532 (69.3)		
3	58 (7.5)		
4‐5	0		
Untreated PD, n (%)	97 (12.6)		
LEDD (treated patients only), mean (SD)	327 (196)	n/a	n/a
Treated participants were on the following medications, n (%)^a^:		n/a	n/a
Levodopa	418 (62.6)		
Dopamine agonist	238 (35.5)		
MAOB‐I	194 (28.9)		
First‐degree relatives with PD, n (%)	114 (14.8)	98 (100)	0
Second‐degree relatives with PD, n (%)	67 (8.7)	16 (16.3)	7 (2.4)
Ever smoked	314 (41.0)	40 (40.8)	126 (44.1)
Number of vascular risk factors^b^, n (%)			
0	360 (47.0)	63 (64.3)	151 (52.8)
1	207 (27.0)	23 (23.5)	69 (24.1)
>2	199 (26.0)	12 (12.2)	66 (23.1)
On medication for, n (%):			
Depression	87 (11.3)	9 (9.2)	22 (7.7)
RBD	7 (0.9)	0	0
Urinary symptoms	57 (7.4)	2 (2.0)	9 (3.1)
Erectile dysfunction (men only)	9 (1.8)	0	1 (0.7)
Constipation	154 (20.2)	8 (8.3)	16 (5.6)

a Percentages relate to the number on each drug; some patients are on more than one class of drug.

b Includes angina, heart failure, stroke or transient ischemic attack, heart attack, diabetes, hypercholesterolemia, and hypertension.

MAOB‐I, monoamine oxidase B inhibitor; n/a, not applicable.

### Genetic Testing

All participants were screened for G2019S and R1441C LRRK2 gene mutations and N370S and L444P GBA gene mutations using methodology described elsewhere.^14^, ^15^

### Clinical Assessment

Validated questionnaires were employed to assess NMS across: (1) neuropsychiatric (Montreal Cognitive Assessment [MoCA]; Beck Depression Inventory‐II [BDI]; Leeds Anxiety and Depression Scale; and the shortened Questionnaire for Impulsive‐Compulsive Disorders in Parkinson's Disease); (2) gastrointestinal (Honolulu‐Asia Ageing Study Constipation Questionnaire); (3) autonomic (lying then standing blood pressure after 2 minutes); (4) sensory (the 16‐stick Sniffin odor identification test); (5) sleep (Epworth Sleepiness Scale; Rapid Eye Movement Sleep Behavior Disorder Screening Questionnaire [RBDSQ]); and (6) sexual function (National Institute of Neurological Disorders and Stroke [NINDS] Parkinson's tool). The International Parkinson and Movement Disorder Society‐revised UPDRS (MDS‐UPDRS I) was also used to assess neuropsychiatric, gastrointestinal, autonomic, sensory, and sleep symptoms in the PD group only. Details of the tests and thresholds for positive symptoms are shown in Supporting Table 2.

**Table 2:** Comparisons of NMS by subject group

	PD	At‐Risk (PD Relatives)	Controls	PD vs. Controls (OR [95% CI]; *P* Value)^a^	At‐Risk vs. Controls (OR [95% CI]; *P* Value)^a^
Neuropsychiatric					
Cognition (MoCA), median (interquartile range)	25 (23‐27)	27 (25‐29)	27 (25‐29)		
‐ Normal cognition, n (%)	534 (70.6)	90 (93.8)	249 (87.7)	2.66 (1.79‐3.96); <0.001^b^	0.70 (0.28‐1.76); 0.45^b^
‐ Possible mild cognitive impairment, n (%)	115 (15.2)	4 (4.2)	25 (8.8)		
‐ Possible dementia, n (%)	107 (14.2)	2 (2.1)	10 (3.5)		
Depression (BDI‐II), median (interquartile range)	8 (4‐12)	4 (2‐8)	4 (1‐7)		
‐ Minimal, n (%)	586 (82.7)	86 (90.5)	261 (93.9)	3.63 (2.12‐6.20); <0.001^b^	1.46 (0.62‐3.43); 0.38^b^
‐ Mild, n (%)	76 (10.7)	5 (5.3)	8 (2.9)		
‐ Moderate, n (%)	39 (5.5)	3 (3.2)	9 (3.2)		
‐ Severe, n (%)	8 (1.1)	1 (1.1)	0 (0)		
Anxiety (Leeds Anxiety and Depression Scale), median (interquartile range)	3 (1‐5)	2 (0‐4)	2 (0‐3)		
‐ Positive screen for anxiety, n (%)	130 (17.3)	7 (7.2)	17 (6.0)	4.08 (2.38‐7.00); <0.001	1.02 (0.40‐2.56); 0.97
ICBs, (QUIP‐S)^c^, n (%)	163 (22.2)	24 (25.8)	62 (22.2)	1.07 (0.76‐1.51); 0.76	0.95 (0.54‐1.66); 0.85
Gastrointestinal					
Constipation (Honolulu Ageing Study), n (%)	375 (49.2)	28 (28.9)	98 (34.2)	1.86 (1.39‐2.50); <0.001	0.93 (0.56‐1.56); 0.80
Autonomic					
Postural drop in systolic blood presure, mmHg, mean (SD)	6.8 (16.0)	−2.2 (14.0)	0.1 (12.5)		
Orthostatic hypotension, n (%)	169 (22.1)	9 (9.2)	19 (6.7)	3.46 (2.10‐5.72); <0.001	1.91 (0.82‐4.45); 0.13
					
Sensory					
Pain (EQ5D), n (%)	425 (55.6)	40 (41.2)	100 (35.0)	2.53 (1.90‐3.38); <0.001^b^	1.45 (0.90‐2.34); 0.13^b^
Hyposmia (Sniffin), n (%)	605 (82.4)	14 (14.4)	37 (13.2)	29.3 (19.7‐43.5); <0.001	1.20 (0.61‐2.34); 0.60
Sleep					
RBD^d^ (RBDSQ), median (interquartile range)	4 (2‐7)	3 (1‐4)	2 (1‐4)		
‐Positive screen for RBD, n (%)	253 (33.5)	17 (18.1)	42 (15.2)	2.67 (1.85‐3.86); <0.001	1.18 (0.63‐2.22); 0.60
Daytime somnolence (ESS)^e^, median (interquartile range)	7 (4‐10)	5 (3‐7)	5 (3‐8)		
‐Positive screen for daytime somnolence, n (%)	173 (22.9)	9 (9.5)	29 (10.2)	2.36 (1.54‐3.61); <0.001	1.01 (0.46‐2.24); 0.98
Treatments					
Number of participants with moderate or severe depression on medication, n treated/n positive (%)	13/47 (27.7)	2/4 (50)	4/9 (44.4)		
Number of participants positive for RBD on medication, n treated/n positive (%)	5/253 (1.9)	0/17 (0)	0/42 (0)		
Number of participants with moderate or severe urinary symptoms on medication, n treated/n positive (%)	6/23 (12.5)	n/a	n/a		
Number of participants with poor or worse erectile function on medication, n treated/n positive (%)	6/209 (2.9)	n/a	n/a		
Number of participants with moderate or severe constipation symptoms on medication, n treated/n positive (%)	59/90 (65.6)	n/a	n/a		

a Adjusted for age and gender.

b OR per unit change using ordinal logistic regression.

c Questionnaire for Impulsive‐Compulsive Disorders in Parkinson's Disease.

d Rapid Eye Movement Sleep Behaviour Disorder.

e Epworth Sleepiness Scale.

n/a, not applicable.

Motor function, disability, and HRQoL were assessed using MDS‐UPDRS III, H & Y staging, Purdue Peg Board Test, Flamingo Balance Test, Schwab and England (S&E) Activities of Daily Living (ADL) scale, and EuroQol‐5 Dimension (EQ‐5D) questionnaire. We classified PD patients into three motor phenotypes (postural instability and gait difficulty [PIGD], tremor dominant [TD], and indeterminate) based on their MDS‐UPDRS motor score.^16^

### Statistical Analysis

Continuous demographic variables were compared using analysis of variance (ANOVA) or Kruskall‐Wallis’ tests (if variances were unequal). The chi‐squared test was used for categorical data. See Supporting Table 2 for details of how the variables were parameterized. For the RBDSQ, we used a threshold of ≥6 for a positive screen in the PD group and ≥5 in relatives and control groups. Use of differing thresholds accounts for all the PD group scoring a point for the presence of a neurological disorder. Logistic regression models, adjusting for age and gender, were used to compare the frequency of NMS in the PD, at‐risk, and control groups. Ordinal logistic regression was used for ordinal outcomes (MOCA, BDI, constipation, pain, and MDS‐UPDRS scores) if Wald's test of parallel lines assumptions was satisfied. Treated and drug‐naïve participants were compared, as well as the different motor phenotypes, using a multivariable logistic regression model adjusting for age, gender, disease duration from diagnosis, levodopa equivalent daily doses^17^ (LEDDs; for the motor phenotypes) and MDS‐UPDRS III. Missing data were excluded from the analysis.

We examined whether NMS were associated with HRQoL (the EQ‐5D summary index score) and disability (S&E) in PD subjects. Owing to non‐normality, we categorized each of these outcomes into quintiles and used ordinal logistic regression to calculate the odds ratio (OR) for a unit change in the outcome, adjusting for age, gender, and disease duration from diagnosis. To try and identify what effect was owing to the NMS component, rather than motor features, the MDS‐UPDRS III motor score was added as a covariate.

To compare NMS with the MDS‐UPDRS motor score, the total number of NMS assessed in the PD group (maximum 19 symptoms, omitting erectile dysfunction as male only) and the total MDS‐UPDRS III motor score were each standardized to a normal distribution with a mean of zero and standard deviation (SD) of 1 before inclusion in the regression model. We used a threshold of 0.05 as a level of significance, but owing to multiple testing, *P* values between 0.05 and 0.001 should be interpreted with caution given that they may reflect a type I error.

## Results

**Figure 1:**
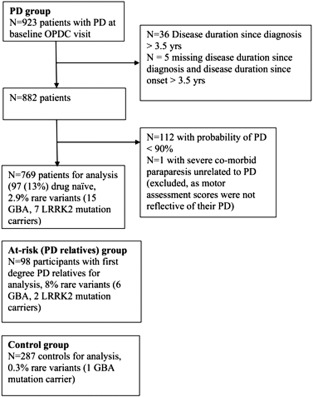
Flow diagram of participants included in analysis. OPDC, Oxford Parkinson's Disease Center.

Baseline data from 1,154 participants (769 PD, 98 at‐risk, and 287 controls) were included (Fig. 1). Basic demographics are shown in Table 1. For all variables, the percentage of missing data was small (≤4%), except for depression and erectile dysfunction, which were higher at 6%.

### Early PD Compared to At‐Risk and Control Groups

The PD group was older than controls (*P* < 0.001). The at‐risk group was younger than both the control group (*P* < 0.001) and PD group (*P* < 0.001). There were more women in the at‐risk and control groups than the PD group (*P* < 0.001). Laxative use was higher in the PD group, compared to controls (*P* < 0.001) and the at‐risk group (*P* = 0.005). The other demographics and treatment rates were similar.

NMS were very common, with only 7 PD cases (1%) describing no NMS on the MDS‐UPDRS. Distribution of the NMS experienced by the PD group is shown in Supporting Figure 1. In order of frequency, the most common NMS experienced in PD were hyposmia, pain, sleep disturbance, urinary symptoms, and fatigue, each affecting over half of subjects (see Supporting Table 3). Additional sensitivity analysis was performed on the BDI (examining the affective and somatic symptoms of depression separately) with only a minor effect on the results.

**Table 3:** Association of nonmotor and motor symptoms with HRQoL and ADL in the early PD group

	HRQoL^a^ (OR [95% CI]; *P* Value)	ADL^b^ (OR [95% CI]; *P* Value)
NMS		
Neuropsychiatric		
Cognition	1.47 (1.08‐2.00); 0.01	1.06 (0.76‐1.47); 0.74
Depression	6.76 (4.50‐10.2); <0.001	2.67 (1.78‐4.01); <0.001
Anxiety	4.89 (3.34‐7.16); <0.001	2.39 (1.61‐3.55); <0.001
ICBs	1.94 (1.38‐2.71); <0.001	1.74 (1.20‐2.52); 0.004
Hallucinations and psychosis	1.11 (0.74‐1.69); 0.61	1.94 (1.23‐3.07), 0.004
Apathy	2.66 (1.87‐3.78); <0.001	1.83 (1.26‐2.65); 0.002
Fatigue	4.53 (3.29‐6.25); <0.001	2.70 (1.94‐3.76); <0.001
Gastrointestinal		
Constipation problems	1.11 (0.85‐1.46); 0.45	1.24 (0.93‐1.66); 0.15
Chewing and swallowing	2.81 (2.00‐3.96); <0.001	3.31 (2.28‐4.81); <0.001
Autonomic		
Urinary problems	1.95 (1.47‐2.59); <0.001	1.67 (1.23‐2.26); 0.001
Saliva and drooling	1.76 (1.33‐2.32); <0.001	1.90 (1.40‐2.58); <0.001
Orthostatic hypotension	1.24 (0.90‐1.73); 0.19	1.19 (0.84‐1.70); 0.33
Sensory		
Pain and other sensations	13.7 (8.63‐21.9); <0.001	2.58 (1.80‐3.69); <0.001
Hyposmia	0.94 (0.66‐1.34); 0.73	1.09 (0.73‐1.62); 0.67
Sleep		
Sleep disturbance	2.57 (1.89‐3.47); <0.001	1.41 (1.03‐1.93); 0.03
RBD	1.90 (1.43‐2.54); <0.001	1.44 (1.05‐1.96); 0.02
Daytime somnolence	2.11 (1.53‐2.91); <0.001	1.97 (1.39‐2.80); <0.001
Sexual dysfunction		
Sexual dysfunction	1.01 (0.70‐1.45); 0.97	1.54 (1.03‐2.30); 0.03
Erectile dysfunction	1.53 (1.06‐2.21); 0.02	2.08 (1.39‐3.10); <0.001
Total NMS score	2.75 (2.33‐3.24); <0.001	2.00 (1.70‐2.36); <0.001
Motor scores		
MDS‐UPDRS III	1.33 (1.15‐1.55); <0.001	2.03 (1.71‐2.42); <0.001
Flamingo Balance Test	1.72 (1.21‐2.44); 0.002	1.52 (1.05‐2.19); 0.03
Purdue Peg Board Test–Total	1.74 (1.27‐2.39); 0.001	2.28 (1.63‐3.19); <0.001

Adjusted for age, gender, and disease duration.

a As assessed by the EQ‐5D.

b As assessed by S&E scale.

The PD group experienced more NMS than the control group in each symptom assessed (see Table 2), apart from ICB. They also experienced more NMS than the at‐risk group in each symptom, except for impulsive‐compulsive behavior (ICB) and orthostatic hypotension. The at‐risk and control groups were similar across each domain tested. The biggest differences comparing the PD group to the control group were anxiety and hyposmia (OR, >4.0).

The at‐risk group of PD relatives did not show any significant increases across the range of NMS studied, compared to controls. There were no significant differences in NMS profile between PD relatives with and without monogenic variants (LRRK2 and GBA). The relative group was comprised of 60 siblings, 23 children, and 12 with at least one of each. Further analysis of the rates of NMS between controls and each of these relative subgroups found no significant differences.

### Drivers of Quality of Life and ADL in Early PD

Both NMS and motor symptoms significantly worsened HRQoL in early PD subjects (Table 3). PD cases had worse HRQoL scores than controls (OR, 4.1; *P* < 0.001). The biggest drivers of worse HRQoL were pain, depression, anxiety, and fatigue.

A similar pattern of symptoms was associated with worse functional status, as measured on the S&E scale. Though associated with worse HRQoL, sleep disturbance, rapid eye movement behaviour disorder (RBD), apathy, and ICB symptoms were not as strongly associated with disability. Motor function, as assessed by the MDS‐UPDRS III and the Purdue peg‐board test, was associated with both poorer HRQoL and greater disability.

Motor function had a relatively modest effect on the HRQoL, compared to NMS. The total number of NMS experienced (maximum, 19) had a greater effect on HRQoL than motor symptoms, as measured by MDS‐UPDRS III (OR, 2.8 vs. 1.3, respectively; Wald's test; *P* < 0.001), although their effects on ADL were similar (OR, 2.0 vs. 2.0; Wald's test; *P* = 0.89).

### Management of NMS in Early PD

Specific treatment for NMS were uncommon in routine clinical care of PD patients, all of whom were managed by local specialist teams. Drug treatment for depression was only given to 13 of 47 (28%) of PD patients with moderate‐to‐severe depression. Suicidal ideation was expressed by 64 of 709 (9%) patients, although almost all stated that they would not act on these thoughts. Only 6 of 23 (26%) participants with moderate‐to‐severe depression who reported suicidal ideation were on treatment. Importantly, 77 of 709 (11%) of all the PD patients were treated with antidepressants, most of whom (83%) only experienced minimal or mild depressive symptoms.

Treatment for RBD (clonazepam or melatonin) was given to just 2% of patients who screened positive for RBD. Only 13% PD cases with significant urinary symptoms and 3% PD cases with erectile dysfunction were being treated. Treatment rates for constipation were better, with 66% of those with moderate or worse symptoms being treated.

### PD Group: Comparison of NMS With Motor Phenotype

Dividing the groups by motor phenotype (Supporting Table 4), the TD group were younger (*P* = 0.01) with an earlier age of onset (*P* = 0.03), compared to PIGD. H & Y staging (*P* < 0.001), but not MDS‐UPDRS motor score (*P* = 0.83), was higher in the PIGD group. The TD group used less dopaminergic medication than the indeterminate (*P* = 0.002) and PIGD (*P* < 0.001) groups.

Participants with the TD phenotype experienced less cognitive, fatigue, depression, anxiety, swallowing, urinary, excess saliva, pain, sleep, and daytime somnolence symptoms than the PIGD phenotype (Supporting Table 5). The indeterminate and PIGD groups had a similar NMS profile. The TD group experienced less NMS than the PIGD (*P* < 0.001) and indeterminate groups (*P* < 0.001) after adjustment for confounders.

### PD Group: Comparison of NMS in Treated and Untreated Subjects

The untreated group had a shorter disease duration (*P* < 0.001), but other demographic variables did not differ significantly (Supporting Table 6). Differences between the groups included more ICB (OR, 3.6; *P* = 0.002) in the treated group, and possibly more daytime somnolence (OR, 2.5; *P* = 0.01) and psychotic (OR, 11.2; *P* = 0.02) symptoms (Supporting Table 3). Treated participants experienced a mean of 7.1 (SD, 2.8; range, 1‐16; 95% confidence interval [CI]: 6.9‐7.3) different NMS, compared to 5.7 (SD, 2.7; range, 1‐11; 95% CI: 5.1‐6.2) in the untreated group. Adjusting for confounders, the treated group experienced more NMS than the untreated group (*P* = 0.002).

## Discussion

To our knowledge, this is the largest study investigating NMS in early PD to date. We highlight that NMS are a common feature even at the early motor stage of the disease, compared to control subjects. Despite their effects on HRQoL, they are under‐recognized and undertreated.

An increasing number of NMS screening tools have been developed for use in research and clinical practice, including PD‐specific questionnaires (such as the Non‐motor Symptom Questionnaire [NMSQuest]^18^) and domain‐specific questionnaires (such as the PD Sleep Scale^19^). Therefore, cross‐comparison of NMS between different cohorts is often limited by the types of screening tool used. Comparisons with control populations may even underestimate differences between groups, given that treatment rates for some symptoms may be higher in the PD group. We found a similar spectrum of NMS in our PD cohort to that reported in the ICICLE‐PD study,^8^ which also found urinary disturbance, saliva and drooling, hyposmia, and constipation were very common. Anxiety, low mood, and forgetfulness were more prevalent in their cohort. This may be a reflection of the means of assessment (the NMSQuest, which dichotomously rates each symptom as present/absent), whereas severity rating scales were mainly used in this cohort. Replication of similar rates of excessive salivary dribbling (49%), chewing, or swallowing difficulties (20%) in our larger cohort lends weight to previous smaller studies, suggesting an earlier evolution of these symptoms than previously recognized.^8^, ^20^ Our cohort reported pain more frequently than other early PD cohorts.^6^, ^8^, ^21^

Neurodegeneration starts many years before the development of the characteristic motor symptoms.^22^, ^23^, ^24^ However, NMS have low specificity given that many are common in the healthy population.^25^ A prodromal cohort of people with a high risk of conversion to PD would be ideal to study this. Though this study did not delineate differences in NMS between the relatives and control group at this stage of the study, inferences should not yet be drawn as to their value in studying prodromal PD. The size of the at‐risk group was not large and it is possible that a true difference exists, but we lacked statistical power to detect this. Second, it is possible that potential converters were too early in the disease process to have clinically assessable symptoms. This group was much younger than the PD group (many were the younger relatives of PD cases); hence, planned longitudinal follow‐up of the cohort may yet detect the emergence of NMS over time as well as assess their validity as predictive features for subsequent motoric onset.

Although selection of a more homogenous at‐risk group comprising larger numbers of LRRK2 and GBA unaffected carriers is arguably attractive for research, the rare occurrence of these mutations in most Western populations often preclude reasonable use.

Quality of life is affected in early PD, although 23% of participants reported no problems. Our findings demonstrate that the accumulation of NMS appears to have a greater effect on HRQoL than motor symptoms in early PD, with pain and mood symptoms being the biggest drivers. Our results are in line with previous studies demonstrating the dominance of depressive symptoms as a significant driver of reduced HRQoL, even in early PD.^3^, ^26^ Evaluation of HRQoL is complex and we acknowledge that depression may lead to an over‐reporting of nondepressive symptoms in assessment scales. However, if people feel miserable or depressed, then one could argue that these measures appropriately capture that their HRQoL would be affected by these feelings. This study showed that pain and sleep symptoms were important determinants of HRQoL in early PD, replicating findings in a smaller cohort.^26^

Treatment of NMS is an emerging, often problematic area for clinicians. We found major opportunities for treatment in some NMS. For example, RBD is not a benign condition, but rarely treated, often causing significant injury to both the patient and their bed partner. Despite depression being one of the most significant drivers of reduced HRQoL in PD, only 28% screening positive for moderate‐to‐severe depression were on antidepressants. Treatment rates were no higher for depression in the PD group than the control group (OR, 1.47; *P* = 0.157: adjusted for age, gender, and depression), despite the higher prevalence in PD and frequent contact with health services. Though it is possible that some patients were receiving other treatments (such as psychological therapy), medication rates still seem low. Even accepting that screening tools can lack specificity (such as the RBDSQ), one would still expect treatment rates to be higher. The use of such tools may improve both recognition and subsequent treatment in early PD.

Patients with the PIGD phenotype experienced more NMS than the TD phenotype across multiple domains. Poorer cognitive performance found in cross‐sectional studies across non‐TD motor phenotypes^27^, ^28^ has now been replicated in this early PD cohort. Other studies^29^ have also found a differential association of NMS with motor subtype,^8^, ^24^, ^30^, ^31^ even in early untreated PD.^29^ However, a recent study failed to find this association in early untreated PD, which may be a reflection of the assessment methods or the earlier motor stage of patients included in the study.^24^ These data provide further evidence of the heterogeneous nature of early PD and potential mechanistic differences between clinical subtypes, even at the early motor stages of the disease.

Untreated PD subjects had a greater burden of NMS than the control group, in keeping with previous studies, although individual symptoms did not show many differences.^6^, ^24^, ^32^, ^33^ Despite using different assessment methods, a high frequency of sleep, fatigue, and urinary symptoms were each found in untreated patients. Treated subjects experienced more NMS than untreated PD, potentially a reflection of more advanced disease or the unwanted side effects of the medication.

Three major features of our study set us apart from other cohorts: (1) PD‐Discovery is one of few cohorts recruiting controls, providing a unique opportunity to compare patient/relative characteristics with healthy subjects; (2) the breadth of clinical features covered, comparable only to the ParkWest cohort,^34^ enables us to characterize NMS in significant detail using validated tools with predominant severity scales; and (3) our cohort has one of the highest numbers of participants with only two other studies (NINDS‐PD LongTerm Study 1^35^ and DATATOP^36^) reporting larger patient numbers. The inclusion of patients with incident cognitive impairment and possible dementia (often excluded from other studies) is a strength, given that their exclusion may bias the findings to less‐aggressive PD subtypes. Study limitations include that despite adjustment for demographic differences between groups, there may still be residual confounding influencing results. All questionnaires are susceptible to recall bias. Despite the large number of participants, approximately 50% of eligible PD subjects in the Thames Valley did not agree to take part. The potential bias of mobility, comorbidity, cognition, and social constraints among nonparticipants could bias results, though we suspect this would (if anything) result in an underestimate of the true prevalence of NMS. However, we believe that the scope of this otherwise unselected, population‐ascertained cohort is highly representative of early PD; hence, meaningful conclusions on NMS can be made.

## Author Roles

(1) Research Project: A. Conception, B. Organization, C. Execution; (2) Statistical Analysis: A. Design, B. Execution, C. Review and Critique; (3) Manuscript Preparation: A. Writing the First Draft, B. Review and Critique.

F.B.: 1A, 1C, 2A, 2B, 3A

M.L.: 2A, 2B, 2C, 3B

M.R.: 1C, 3B

C.R.: 1C, 3B

K.N.: 1C, 3B

S.G.E.: 1C, 3B

H.R.F.: 1C, 3B

Y.B.‐S.: 1A, 1B, 2B, 2C, 3A

M.T.M.H.: 1A, 1B, 1C, 2C, 3A

## Financial Disclosures

F.B. has been employed by the Oxford Parkinson's Disease Center, UK. M.L. has been employed by the School of Social and Community Medicine, UK. M.R. was funded by the British Research Council, UK. C.R. has been employed by the Oxford Parkinson's Disease Center, UK. K.N. has been employed by Northampton General Hospital, UK. S.G.E. has been employed by the Oxford Parkinson's Disease Center, UK. H.R.F. has been employed by the University of Oxford, UK. Y.B.‐S. has served on advisory boards as a member of the Multiple Sclerosis Risk Sharing Scheme Scientific Advisory Board; has been employed by the University of Bristol; has received royalties from books published by Oxford University Press and Wiley; and has received grants from Parkinson's UK, Cancer Research UK, National Institute of Health Research, British Heart Foundation, and Medical Research Council. M.T.M.H. was funded by the OPDC Monument Discovery award, the Oxford Biomedical Research Center, and the National Institute of Health Research Clinical Research Network.

## Ethics Approval

The study was undertaken with the understanding and written consent of each subject, approval of the local NHS ethics committee, and in compliance with national legislation and the Declaration of Helsinki. Ethical approval for this study was granted by the Berkshire Ethics Committee, South Central, National Research Ethics Service (UK; reference no.: 10/H0505/71).

## Supporting information

Additional Supporting Information may be found in the online version of this article at the publisher's web‐site.

Supplementary Information
