# Supplementary material for: Delineating nonmotor symptoms in early Parkinson's disease and first‐degree relatives
Source: Mov Disord. 2015 Jul 14;30(13):1759–66. doi: 10.1002/mds.26281 (PMC5034839; doi:10.1002/mds.26281)
Supplement: Supplementary file 1 — Supplementary Information [file MDS-30-1759-s001.docx]

# SUPPLEMENTARY MATERIAL - Delineating NMS in Early Parkinson’s Disease and First Degree Relatives. Fahd Baig et al.

### SUPPLEMENTAL TABLE 1 - Recruitment for the PD-Discovery Cohort

| Recruitment | Participants were prospectively recruited from the Thames Valley (UK) population (PD-Discovery, website: http://opdc.medsci.ox.ac.uk).  **Parkinson’s Disease Group:**  Parkinson’s disease (PD) cases were recruited from neurology and elderly care clinics in hospitals based in the following locations: Oxford, Reading, Newbury, Wexham Park, High Wycombe, Aylesbury, Milton Keynes, Northampton, Banbury, Kettering, Poole and Swindon. Neurologists, specialist nurses, geriatricians and GP’s from participating hospitals were asked to identify and refer all cases within 3 years of diagnosis. Additional support from the Dementia and Neurodegenerative Diseases Network (DeNdRoN) helped disseminate information and encourage recruitment. All the participating clinicians are regularly contacted to ensure screening of incident cases. Eligible cases were then contacted by post to invite them to participate in the study.  **At-risk group of first-degree relatives:**  The at-risk group comprised of participants with a first-degree relative carrying a diagnosis of PD, confirmed at the research visit using a numerated family history questionnaire. All first-degree relatives of PD patients included in the study were invited to participate, however relatives of carriers of LRRK2 and GBA mutations were actively recruited for inclusion in other sub-studies. Also, relatives who had more than one affected family member were more likely to volunteer for the study. Therefore, despite their small number, these factors would have led to an over-sampling of these cases.  **Control group:**  The control population were recruited from spouses and friends of patients taking part in the study, as well as the general public. |
| --- | --- |
| Inclusion and Exclusion Criteria | **Parkinson’s Disease Group:**  All patients who were referred were pre-screened for a diagnosis of Parkinson’s disease. Each participant had their diagnosis reviewed by a movement disorders neurologist or fellow with specific training for PD assessment during their clinic visit. All cases satisfying the UK PD Brain Bank Criteria for a diagnosis of PD were eligible for inclusion. Cases reviewed in clinic more than 3.5 years from their diagnosis were excluded (in line with other UK based studies). Cases with more than one relative with a diagnosis of PD who otherwise fulfil the criteria, were still included.  Patients who developed dementia within 12 months of the onset of their symptoms were excluded as having dementia with Lewy-body disease.  Atypical parkinsonian features were screened using the National Institute of Neurological Disorders and Stroke Parkinson’s tool. The physician was then asked to rate the percentage likelihood of PD with all cases excluded from subsequent analysis if they had a <90% probability of PD at their latest clinic visit.  **At-risk group of relatives:**  The at-risk group of relatives were eligible for inclusion if they had a relative with a diagnosis of PD included in the PD-Discovery cohort. They were examined at the clinic visit by a movement disorders neurologist or fellow to exclude a diagnosis of PD.  **Control group:**  The control group were screened for first-degree relatives with PD by use of a numerated family history questionnaire and examined to exclude a diagnosis of PD. |

###

### SUPPLEMENTAL TABLE 2 - Clinical metrics used for assessment of non-motor symptoms, motor symptoms, health related quality of life and activities of daily living

|  | **Scale / Clinical assessment** |
| --- | --- |
| **NON-MOTOR SYMPTOMS (NMS)** |  |
| Movement Disorder Society-revised Unified Parkinson’s Disease Rating Scale Parts I & II | The MDS-UPDRS rates each symptom on a scale of 0-4, each score relating to an absent, slight, mild, moderate or severe symptom. We used the following NMS assessed by MDS-UPDRS I: hallucinations and psychosis; apathy; sleep problems; pain and other sensations; urinary problems; and fatigue. Questions from MDS-UPDRS II assessing dysphagia and hypersialorrhoea were also used as NMS. As our patients are relatively early in the natural history, we dichotomised the results (0 versus, ≥ 1) for analysis.[1] |
| **Neuropsychiatric** |  |
| Depression and anxiety assessment | Leeds Scales for the Self Assessment of Anxiety and Depression: using a threshold of >7 for to score.[2]  Beck Depression Inventory II: using scores of 0-13 to denote minimal depression, scores of 14-19 to denote mild depression, scores of 20-28 denote moderate depression, and scores of 29-63 to denote severe depression.[3] |
| **Cognition** | Montreal Cognitive Assessment tool (MOCA): using screening cut-off scores of 22-23 to denote possible mild cognitive impairment (MCI) and <22 for possible dementia. [4] |
| Impulse control behaviours | The shortened Questionnaire for Impulsive-Compulsive Disorders in Parkinson’s Disease (QUIP-S): using a positive response in any category as a positive screen for impulse control behaviours.[5] |
| **Gastrointestinal** |  |
| Constipation symptoms | The constipation questionnaire as based on the Honolulu-Asia Ageing Study: <1 daily bowel movement or use of laxatives were deemed constipated.[6] The MDS-UPDRS part I question 1.11 was also used to compare constipation symptoms with laxative use. |
| **Sleep** |  |
| REM sleep behaviour disorder | Rapid Eye Movement Sleep Behaviour Disorder Screening Questionnaire (RBDSQ): using a threshold of ≥6 for a positive screen in the early PD group and ≥5 in the relatives and control groups. The use of differing thresholds accounts for all the PD group scoring a point for the presence of a neurological disorder.[7] |
| Daytime somnolence symptom | Epworth Sleepiness Scale (ESS): using a threshold of ≥10 as a positive screen.[8] |
| **Autonomic** |  |
| Orthostatic hypotension assessment | Blood pressure was measured after the patient lies flat for 3 minutes then again after standing upright for 2 minutes: a systolic drop of blood pressure (BP) of ≥20mmHg or a diastolic drop of ≥10mmHg were deemed positive.[9] |
| **Sensory** |  |
| Pain | EQ5D: Question 5: A 3 point scale of no, moderate or extreme pain or discomfort. Moderate or extreme pain were deemed positive.[10] |
| Hyposmia assessment | Sniffin sticks odour identification test dichotomised at 10th percentile according to normal values for age and sex:[11]  Males and females age 16-≤36: 11 or lower;  Males aged >36-≤55: 12 or lower;  females aged >36-≤55: 11 or lower;  Males and females age >55: 9 or lower. |
| **Sexual Dysfunction** |  |
| Sexual dysfunction | Questionnaire from PD-DOC study / NIH common data elements: present or absent.[14] |
| Erectile dysfunction | Questionnaire from PD-DOC study / NIH common data elements: rated very poor, poor, fair, good or very good. Very poor and poor were considered positive for erectile dysfunction.[13] |
| **Oculomotor Dysfunction** |  |
| Oculomotor disturbance | Questionnaire from PD-DOC study / NIH common data elements: present or absent.[14] |
| **Total NMS Score** |  |
| Total NMS score – PD group | The sum total of 19 NMS domains assessed were used, excluding erectile dysfunction as gender specific: cognition; hallucinations and psychosis; apathy; fatigue; anxiety; depression; ICB; orthostatic hypotension; urinary dysfunction; saliva and drooling; chewing and swallowing; constipation; pain; hyposmia; sleep problems; daytime somnolence; RBD; sexual dysfunction; and oculomotor disturbance.  Where a single item was missing from a case, that symptom was imputed as negative and the case included in the total score. |
| Comparison between PD and control groups | The sum total of 9 NMS domains assessed was used: cognition; anxiety; depression; ICB; constipation; orthostatic hypotension; pain; hyposmia; daytime somnolence; and RBD.  Where a single item was missing from a case, that symptom was imputed as negative and the case included in the total score. |
| **MOTOR SYMPTOMS** |  |
| UPDRS part III (motor symptoms score) | The MDS-UPDRS uses a scale of 0-4, each score relating to an absent, mild, moderate or severe motor symptom. The motor score part III examination includes assessments of rigidity, bradykinesia, tremor, gait and postural instability with other features.[1] |
| Purdue Peg Board test (hand dexterity, speed and co-ordination) | The total number of pegs inserted into the board using each hand individually and then both hands over a total of 90s. [12] The data was stratified into quintiles for comparison, with the worst performing quintile considered poor performers. |
| Flamingo balance test | The participant is timed on their ability to stand on one leg for up to 30s.[13] The data was stratified into quintiles for comparison, with the worst performing quintile considered poor performers. |
| **ACTIVITIES OF DAILY LIVING** |  |
| Schwab and England ADL scale | Participants are asked to rate their ability to manage their ADL on an 11 point scale from essentially normal to a bedridden state. |
| **QUALITY OF LIFE** |  |
| EQ-5D-3L | The 5 domains of mobility, self-care, usual activities, pain and mood each on a 3 point scale which is then converted into a summary index score for comparison.[10] The summary index was then stratified into quintiles for analysis. |

1. Goetz CG, Tilley BC, Shaftman SR, et al. Movement Disorder Society-Sponsored Revision of the Unified Parkinson's Disease Rating Scale (MDS-UPDRS): Scale Presentation and Clinimetric Testing Results. *Mov. Disord.* 2008;**23**(15):2129-70.

2. Snaith RP, Bridge GW, Hamilton M. The Leeds scales for the self-assessment of anxiety and depression. *Br. J. Psychiatry* 1976;**128**:156-65.

3. Beck AT, Ward C, Mendelson M. Beck depression inventory (BDI). *Arch. Gen. Psychiatry* 1961;**4**(6):561-71.

4. Hu MT, Szewczyk-Krolikowski K, Tomlinson P, et al. Predictors of cognitive impairment in an early stage Parkinson's disease cohort. *Mov. Disord.* 2014

5. Weintraub D, Hoops S, Shea JA, et al. Validation of the questionnaire for impulsive-compulsive disorders in Parkinson's disease. *Mov. Disord.* 2009;**24**(10):1461-7.

6. Abbott RD, Ross GW, White LR, et al. Environmental, life-style, and physical precursors of clinical Parkinson's disease: recent findings from the Honolulu-Asia Aging Study. *J. Neurol.* 2003;**250 Suppl 3**:III30-9.

7. Nomura T, Inoue Y, Kagimura T, et al. Utility of the REM sleep behavior disorder screening questionnaire (RBDSQ) in Parkinson's disease patients. *Sleep Med.* 2011;**12**(7):711-3.

8. Johns MW. A new method for measuring daytime sleepiness: the Epworth sleepiness scale. *Sleep* 1991;**14**(6):540-5.

9. Freeman R, Wieling W, Axelrod FB, et al. Consensus statement on the definition of orthostatic hypotension, neurally mediated syncope and the postural tachycardia syndrome. *Auton. Neurosci.* 2011;**161**(1-2):46-8.

10. EuroQol G. EuroQol--a new facility for the measurement of health-related quality of life. *Health Policy* 1990;**16**(3):199-208.

11. Hummel T, Kobal G, Gudziol H, et al. Normative data for the "Sniffin' Sticks" including tests of odor identification, odor discrimination, and olfactory thresholds: an upgrade based on a group of more than 3,000 subjects. *Eur. Arch. Otorhinolaryngol.* 2007;**264**(3):237-43.

12. Vingerhoets FJ, Schulzer M, Calne DB, et al. Which clinical sign of Parkinson's disease best reflects the nigrostriatal lesion? *Ann. Neurol.* 1997;**41**(1):58-64.

13. Tsigilis N, Douda H, Tokmakidis SP. Test-retest reliability of the Eurofit test battery administered to university students. *Percept. Mot. Skills* 2002;**95**(3 Pt 2):1295-300.

14. PD-DOC study / NIH common data elements: http://grants.nih.gov/grants/guide/rfa-files/RFA-NS-11-001.html. Date accessed 08/12/2015.

At the date this paper was written, URLs or links referenced herein were deemed to be useful supplementary material to this paper. Neither the author nor the journal warrants or assumes liability for the content or availability of URLs referenced in this paper.

### SUPPLEMENTAL TABLE 3 – Summary of all PD cases and comparison of non-motor symptoms in Treated vs Untreated PD

|  | **All PD cases**  (n=769) | **Treated PD**  (n=672) | **Untreated PD**  (n=97) | **Treated vs Untreated PD** (Odds Ratio (95% CI))* | **Treated vs Untreated** **PD** (p value for odds ratio) |
| --- | --- | --- | --- | --- | --- |
| **Neuropsychiatric** |  |  |  |  |  |
| **Cognition** (Montreal Cognitive Assessment) (median(interquartile range)) | 25(23-27) | 25(23-27) | 25(23-28) |  |  |
| - Normal Cognition (n(%)) | 534(70.6) | 470(70.9) | 64(68.8) | 0.92(0.56-1.51)^$^ | p=0.74 |
| - Possible Mild Cognitive Impairment (n(%)) | 115(15.2) | 96(14.5) | 19(20.4) |  |  |
| - Possible dementia (n(%)) | 107(14.2) | 97(14.6) | 10(10.8) |  |  |
| **Depression** (Beck’s Depression Inventory) (median(interquartile range))^b^ | 8(4-12) | 8(4-12) | 7(3-10) |  |  |
| - Minimal (n(%)) | 586(82.7) | 508(82.2) | 78(85.7) | 1.09(0.57-2.10)^$^ | p=0.79 |
| - Mild (n(%)) | 76(10.7) | 69(11.2) | 7(7.7) |  |  |
| - Moderate (n(%)) | 39(5.5) | 34(5.5) | 5(5.5) |  |  |
| - Severe (n(%)) | 8(1.1) | 7(1.1) | 5(1.1) |  |  |
| **Anxiety** (Leeds Anxiety and Depression Scale) (median(interquartile range))^c^ | 3(1-5) | 3(1-5) | 2(0.5-4) |  |  |
| - Anxiety positive screen (n(%)) | 130(17.3) | 121(18.5) | 9(9.4) | 1.91(0.91-4.0) | p=0.09 |
| **Impulse Control Behaviours** (QUIP-S)^a^ (n(%))^a^ | 163(22.2) | 156(24.3) | 7(7.6) | 3.64(1.58-8.38) | p=0.002 |
| **Hallucinations & psychosis** (MDS-UPDRS)^b^ (n(%)) | 91(11.9) | 89(13.3) | 2(2.1) | 11.2(1.51-82.3)^$^ | p=0.02 |
| **Apathy** (MDS-UPDRS) ^b^ (n(%)) | 143(18.6) | 128(19.1) | 15(15.6) | 1.24(0.67-2.28) | p=0.49 |
| **Fatigue** (MDS-UPDRS) ^b^ (n(%)) | 531(69.8) | 472(71.0) | 59(61.5) | 1.31(0.81-2.10) | p=0.27 |
| **Gastrointestinal** |  |  |  |  |  |
| **Constipation** (Honolulu Ageing Survey) (n(%)) | 375(49.2) | 334(50.2) | 41(42.7) | 1.34(0.85-2.14) | p=0.21 |
| **Chewing and swallowing** (MDS-UPDRS) ^b^ (n(%)) | 155(20.3) | 139(20.8) | 16(16.7) | 0.98(0.53-1.79) | p=0.94 |
| **Autonomic** |  |  |  |  |  |
| **Urinary problems** (MDS-UPDRS) ^b^ (n(%)) | 478(62.7) | 416(62.5) | 62(64.6) | 0.87(0.57-1.32) ^$^ | p=0.50 |
| **Saliva and drooling** (MDS-UPDRS) ^b^ (n(%)) | 373(48.9) | 335(50.2) | 38(39.6) | 1.39(0.89-2.17) | p=0.14 |
| Postural drop in systolic BP (mmHg)(mean(sd)) | 6.8(16.0) | 7.2(16.3) | 4.3(13.3) |  |  |
| **Orthostatic hypotension** (n(%)) | 169(22.1) | 156(23.3) | 13(13.4) | 1.85(0.95-3.58) ^$^ | p=0.07 |
| **Sensory** |  |  |  |  |  |
| **Pain and other sensations** (MDS-UPDRS) ^b^ (n(%)) | 592(77.7) | 529(79.4) | 63(65.6) | 1.58(1.03-2.42) ^$^ | p=0.04 |
| **Hyposmia** (Sniffin) (n(%)) | 605(82.4) | 532(82.9) | 73(79.4) | 1.45(0.81-2.61) | p=0.21 |
| **Sleep** |  |  |  |  |  |
| **Sleep disturbance** (MDS-UPDRS) ^b^ (n(%)) | 528(69.3) | 470(70.6) | 58(60.4) | 1.54(1.03-2.31) ^$^ | p=0.04 |
| **REM sleep behaviour disorder** (RBDSQ)^c^(median(inter-quartile range)) | 4(2-7) | 4(2-7) | 3(2-5) |  |  |
| - Positive screen for RBD (n(%)) | 253(33.5) | 230(34.9) | 23(24.0) | 1.61(0.96-2.71) | p=0.07 |
| **Daytime somnolence** (ESS)^d^(median(inter-quartile range)) | 7(4-10) | 7(4-10) | 5(3-8) |  |  |
| - Positive screen for daytime somnolence (n(%)) | 173(22.9) | 163(24.8) | 10(10.3) | 2.52(1.25-5.07) | p=0.01 |
| **Sexual Dysfunction** |  |  |  |  |  |
| **Sexual dysfunction** (n(%)) | 138(18.4) | 124(18.9) | 14(14.7) | 1.44(0.76-2.75) | p=0.27 |
| **Erectile dysfunction** (n(%)) | 209(43.7) | 188(45.3) | 21(33.3) | 1.20(0.72-1.99) ^$^ | p=0.48 |
| **Miscellaneous** |  |  |  |  |  |
| **Oculomotor Dysfunction** (n(%)) | 17(2.2) | 16(2.4) | 1(1.1) | 2.89(0.37-22.8) | p=0.31 |

*Adjusted for age, gender, disease duration (from diagnosis) and UPDRS III motor score. ^$^ Odds ratio per unit change using ordinal logistic regression.^a^ Questionnaire for Impulsive-Compulsive Disorders in Parkinson’s Disease. ^b^ Movement Disorder Society-revised Unified Parkinson’s Disease Rating Scale. ^c^ Rapid Eye Movement Sleep Behaviour Disorder. ^d^ Epworth Sleepiness Scale

### SUPPLEMENTAL TABLE 4 - Demographics of PD group divided by phenotype

| **Basic Demographics** | **Tremor Dominant** (n=407) | I**ndeterminate** (n=95) | **PIGD**  (n=254) |
| --- | --- | --- | --- |
| Age (mean, range (SD)) | 67.1, 37-87(9.4) | 66.9, 43-86(9.3) | 69.0, 32-89(9.9) |
| Female n(%) | 120(29.5) | 37(39.0) | 100(39.4) |
| Ethnicity (non-white n(%)) | 5(1.2) | 2(2.1) | 4(1.6) |
| Age of motoric symptom onset (mean, range (SD)) | 64.3, 28-85(9.6) | 64.0, 41-84(9.6) | 66.0, 31-87(9.9) |
| Disease duration from symptom onset (mean, range (SD)) | 2.9, 0.2-13.2(1.9) | 2.8, 0.2-13.9(1.9) | 3.1, 0.4-11.4(1.7) |
| Disease duration from diagnosis (mean, (SD)) | 1.27(1.0) | 1.34(1.0) | 1.40(0.9) |
| MDS-UPDRS III (mean(SD)) | 26.2(10.4) | 26.1(11.6) | 26.7(11.7) |
| Hoehn and Yahr Stage (n(%))  0  1  2  3  4-5 | 0  111(27.3)  287(70.5)  9(2.2)  0 | 0  22(23.2)  68(71.6)  5(5.3)  0 | 0  42(16.5)  168(66.1)  44(17.3)  0 |
| Untreated PD (n(%)) | 72(17.7) | 6(6.3) | 17(6.8) |
| Levodopa equivalent daily dosage (mean(SD)) | 239(199) | 310(205) | 353(221) |
| Participants on the following medications (n(%)):  Levodopa  Dopamine agonist  MAOB-I | 186(45.9)  116(28.5)  109(26.8) | 57(60.0)  36(37.9)  24(25.3) | 170(67.5)  82(32.4)  55(21.7) |
| Ever smoked (n(%)) | 153(37.8) | 45(47.4) | 110(43.3) |
| Number of vascular risk factors* (n(%))  0  1  >2 | 188(46.3)  114(28.1)  104(25.6) | 47(49.5)  27(28.4)  21(22.1) | 116(45.9)  65(25.7)  72(28.5) |

*Includes angina, heart failure, stroke or TIA, heart attack, diabetes, hypercholesterolaemia and hypertension.

### SUPPLEMENTAL TABLE 5 – Comparison of non-motor symptoms by motor phenotype

|  | **Tremor Dominant (TD)** (n=407) | **Indeterminate** (n=95) | **Postural Instability Gait Difficulty (PIGD)**  (n=254) | **PIGD vs TD** (Odds Ratio (95% CI), p value)* | **PIGD vs Indeterminate** (Odds Ratio (95% CI), p value)* | **Indeterminate vs TD**  (Odds Ratio (95% CI), p value)* |
| --- | --- | --- | --- | --- | --- | --- |
| **Neuropsychiatric** |  |  |  |  |  |  |
| **Cognition** (Montreal Cognitive Assessment) (median(interquartile range)) | 26(24-28) | 26(23-27) | 25(22-27) |  |  |  |
| - Normal Cognition (n(%)) | 305(76.8) | 64(68.8) | 159(62.9) | 1.88(1.27-2.69), p=0.001^$^ | 0.98 (0.58-1.66), p=0.94^$^ | 1.88(1.12-3.17), p=0.02^$^ |
| - Possible Mild Cognitive Impairment (n(%)) | 50(12.6) | 10(10.8) | 51(20.2) |  |  |  |
| - Possible dementia (n(%)) | 42(10.6) | 19(20.4) | 43(17.0) |  |  |  |
| **Depression** (Beck’s Depression Inventory) (median(interquartile range))^b^ | 6(3-10) | 9(5-13) | 9(6-13) |  |  |  |
| - Minimal (n(%)) | 330(86.8) | 68(79.1) | 181(76.7) | 2.21 (1.41-3.47), p=0.001^$^ | 1.24(0.67-2.29), p=0.50^$^ | 1.79(0.96-3.31), p=0.065^$^ |
| - Mild (n(%)) | 35(9.2) | 11(12.8) | 30(12.7) |  |  |  |
| - Moderate (n(%)) | 13(3.4) | 6(7.0) | 20(8.5) |  |  |  |
| - Severe (n(%)) | 2(0.5) | 1(1.2) | 5(2.1) |  |  |  |
| **Anxiety** (Leeds Anxiety and Depression Scale) (median(interquartile range))^c^ | 2(1-4) | 4(1-6) | 3(1-6) |  |  |  |
| - Anxiety positive screen (n(%)) | 50(12.4) | 19(20.7) | 60(24.3) | 2.15(1.38-3.36), p=0.001 | 1.27(0.70-2.32), p=0.43 | 1.69(0.92-3.10), p=0.09 |
| **Impulse Control Behaviours** (QUIP-S)(n(%))^a^ | 76(19.6) | 29(31.9) | 57(23.2) | 1.22(0.79-1.87), p=0.37 | 0.66(0.37-1.18), p=0.16 | 1.84(1.05-3.20), p=0.03 |
| **Hallucinations & psychosis** (MDS-UPDRS)^b^ (n(%)) | 38(9.4) | 13(13.7) | 39(15.4) | 1.63(0.98-2.72), p=0.06^$^ | 1.17(0.58-2.37), p=0.66^$^ | 1.39(0.69-2.80), p=0.36^$^ |
| **Apathy** (MDS-UPDRS) ^b^ (n(%)) | 71(17.4) | 22(23.2) | 49(19.3) | 1.14(0.74-1.76), p=0.55 | 0.83(0.46-1.48), p=0.52 | 1.38(0.79-2.42), p=0.25 |
| **Fatigue** (MDS-UPDRS) ^b^ (n(%)) | 252(62.1) | 74(77.9) | 200(79.1) | 2.31(1.57-3.40), p<0.001 | 1.07(0.60-1.92), p=0.82 | 2.15(1.26-3.69), p=0.005 |
| **Gastrointestinal** |  |  |  |  |  |  |
| **Constipation** (Honolulu Ageing Survey) (n(%)) | 196(48.3) | 37(39.4) | 136(53.8) | 1.13(0.80-1.58), p=0.49 | 1.72(1.05-2.84), p=0.03 | 0.66(0.41-1.06), p=0.09 |
| **Chewing and swallowing** (MDS-UPDRS) ^b^ (n(%)) | 52(12.8) | 20(21.1) | 82(32.3) | 3.44(2.25-5.26), p<0.001^$^ | 1.71(1.04-2.81), p=0.04^$^ | 2.04 (1.12-3.72), p=0.02^$^ |
| **Autonomic** |  |  |  |  |  |  |
| **Urinary problems** (MDS-UPDRS) ^b^ (n(%)) | 237(58.2) | 65(69.2) | 172(67.7) | 2.00(1.46-2.73), p<0.001^$^ | 1.07(0.68-1.69), p=0.76^$^ | 1.77(1.16-2.70), p=0.008^$^ |
| **Saliva and drooling** (MDS-UPDRS) ^b^ (n(%)) | 180(44.2) | 48(50.5) | 143(56.3) | 1.63(1.19-2.24), p=0.002^$^ | 1.25(0.80-1.98), p=0.33^$^ | 1.30(0.84-2.02), p=0.24^$^ |
| Postural drop in systolic BP (mmHg)(mean(sd)) | 5.9(15.9) | 7.6(16.6) | 7.8(15.4) |  |  |  |
| **Orthostatic hypotension** (n(%)) | 77(19.0) | 25(26.3) | 65(25.7) | 1.42(0.94-2.13), p=0.01 | 0.82(0.47-1.45), p=0.50 | 1.72(0.99-2.97), p=0.05 |
| **Sensory** |  |  |  |  |  |  |
| **Pain and other sensations** (MDS-UPDRS) ^b^ (n(%)) | 299(73.5) | 71(75.5) | 216(85.0) | 2.34(1.71-3.21), p<0.001^$^ | 1.42(0.89-2.25), p=0.14^$^ | 1.66(1.07-2.59), p=0.03^$^ |
| **Hyposmia** (Sniffin) (n(%)) | 319(82.0) | 77(81.9) | 199(82.9) | 1.21(0.77-1.90), p=0.40 | 1.12(0.59-2.12), p=0.73 | 1.08(0.59-1.97), p=0.80 |
| **Sleep** |  |  |  |  |  |  |
| **Sleep disturbance** (MDS-UPDRS) ^b^ (n(%)) | 266(65.4) | 68(72.3) | 188(74.0) | 1.61(1.19-2.17), p=0.002^$^ | 0.96 (0.62-1.49), p=0.86^$^ | 1.68(1.11-2.54), p=0.02^$^ |
| **REM sleep behaviour disorder** (RBDSQ)^c^(median(inter-quartile range)) | 4(2-6) | 4(3-6) | 4(3-7) |  |  |  |
| - Positive screen for RBD (n(%)) | 121(30.0) | 35(37.6) | 93(37.1) | 1.42(1.00-2.03), p=0.05 | 1.01(0.61-1.68), p=0.98 | 1.41(0.87-2.29), p=0.17 |
| **Daytime somnolence** (ESS)^d^(median(inter-quartile range)) | 6(4-9) | 8(5-11) | 8(5-11) |  |  |  |
| - Positive screen for daytime somnolence (n(%)) | 68(16.9) | 30(32.3) | 72(28.8) | 1.88(1.26-2.82), p=0.002 | 0.83(0.49-1.42), p=0.50 | 2.26(1.33-3.83), p=0.002 |
| **Sexual Dysfunction** |  |  |  |  |  |  |
| **Sexual dysfunction** (n(%)) | 73(18.2) | 14(15.1) | 46(18.8) | 1.13(0.72-1.76), p=0.60 | 1.37(0.69-2.72), p=0.37 | 0.82(0.43-1.59), p=0.56 |
| **Erectile dysfunction** (n(%)) | 111(40.8) | 17(30.4) | 78(54.9) | 1.18(0.80-1.74), p=0.39^$^ | 1.76(0.99-3.13), p=0.05^$^ | 0.67(0.39-1.15), p=0.15^$^ |
| **Miscellaneous** |  |  |  |  |  |  |
| **Oculomotor Dysfunction** (n(%)) | 9(2.2) | 4(4.2) | 4(1.6) | 0.58(0.16-2.03), p=0.39 | 0.40(0.09-1.70), p=0.22 | 1.44(0.41-5.04), p=0.57 |

*Adjusted for age, gender, disease duration from diagnosis, levodopa equivalent daily dosage and UPDRS III motor score. ^$^ Odds ratio per unit change using ordinal logistic regression.^a^ Questionnaire for Impulsive-Compulsive Disorders in Parkinson’s Disease. ^b^ Movement Disorder Society-revised Unified Parkinson’s Disease Rating Scale. ^c^ Rapid Eye Movement Sleep Behaviour Disorder. ^d^ Epworth Sleepiness Scale.

### SUPPLEMENTAL TABLE 6 – Demographics of Treated and Untreated PD group

| **Basic Demographics** | **Treated PD group** (n=672) | **Untreated PD group** (n=97) |
| --- | --- | --- |
| Age (mean, range (SD)) | 67.7, 37-89 (9.6) | 67.4, 32-84 (9.3) |
| Gender (female n(%)) | 234(34.8) | 27(27.8) |
| Ethnicity (non-white n(%)) | 10(1.5) | 1(1.0) |
| Age of motoric symptom onset (mean, range (SD)) | 64.7, 28-87 (9.8) | 65.1, 31-82 (9.3) |
| Disease duration from symptom onset in years (mean, range (SD)) | 3.0, 0.2-13.9 (1.9) | 2.2, 0.2-6.8 (1.4) |
| Disease duration from diagnosis in years (mean, (SD)) | 1.4 (1.0) | 0.8 (0.8) |
| MDS-UPDRS III (mean(SD)) | 26.6 (11.0) | 24.8(10.5) |
| Hoehn and Yahr Stage (n(%))  0  1  2  3  4-5 | 0  147(21.9)  472(70.2)  53(7.9)  0 | 0  31(32.3)  60(62.5)  5(5.2)  0 |
| LEDD (mean(SD)) | 327 (196) | n/a |
| Participants on the following medictions (n(%)):  Levodopa  Dopamine agonist  MAOB-I | 418(62.6)  238(35.5)  194(28.9) | n/a |
| First degree relatives with PD (n(%)) | 97(14.4) | 17(17.5) |
| Second degree relatives with PD (n(%)) | 63(9.4) | 4(4.1) |
| Ever smoked | 276(41.2) | 40(41.7) |
| Number of vascular risk factors*(n(%))  0  1  >2 | 317(47.3)  184(27.5)  169(25.2) | 43(44.8)  23(24.0)  30(31.3) |

### LEGEND - SUPPLEMENTAL FIGURE 1

This graph shows the percentage frequency of the total number of non-motor symptoms experienced by participants with early Parkinson’s Disease (n=746).
